# A Novel Technique of Spectral Discrimination of Variants of Sickle Cell Anemia

**DOI:** 10.1155/2018/5942368

**Published:** 2018-08-27

**Authors:** Vadivel Masilamani, Sandanasamy Devanesan, Fatma AlQathani, Mashael AlShebly, Hebatullah Hassan Daban, Duran Canatan, Karim Farhat, Mansour Jabry, Mohamad S. AlSalhi

**Affiliations:** ^1^Department of Physics and Astronomy, College of Science, King Saud University, Riyadh, Saudi Arabia; ^2^Research Chair in Laser Diagnosis of Cancers, Department of Physics and Astronomy, College of Science, King Saud University, Riyadh, Saudi Arabia; ^3^Hematology Unit, Department of Pathology, College of Medicine, King Saud University and King Saud University Medical City, Riyadh, Saudi Arabia; ^4^Department of Obstetrics and Gynecology, College of Medicine, King Khalid University Hospital, King Saud University, Riyadh, Saudi Arabia; ^5^Department of Physics and Astronomy, College of Science, King Saud University (Ladies Section), Riyadh, Saudi Arabia; ^6^Hemoglobinopathy Diagnosis Center of Mediterranean Blood Diseases Foundation, Antalya, Turkey; ^7^Cancer Research Chair, College of Medicine, King Saud University, Riyadh 11451, Saudi Arabia

## Abstract

Sickle cell anemia (SCA) is an inherited blood disorder with worldwide incidence of 15%; out of this, it is found in up to 20% in countries like Kingdom of Saudi Arabia and Bahrain. The standard conventional method of detection is complete blood count (CBC) followed by hemoglobin electrophoresis or high-performance liquid chromatography (HPLC) or both. In this context, spectral detection of variants of sickle cell anemia (SCA) is an innovative technique, which when made accurate and reliable could be an effective alternative, since the instrumentation is compact (5 kg) and hence portable. This makes mass screening even in remote villages possible. In this paper, we give the essential aspects of fluorescent spectral features of sickle cell trait (SCT), sickle cell disease (SCD), beta (*β*) thalassemia trait (BTT) + SCD, and beta (*β*) thalassemia disease (BTD) + SCD. All the above four major variants could be discriminated among themselves and also from the normal control blood sample. All these analyses could be carried out with 5 ml of blood, in a time period of 10 minutes. The results of this paper give strong support for an alternative method, a spectral technique, for molecular-level diagnosis of sickle cell anemia and other closely related blood disorders.

## 1. Introduction

Red blood cell (RBC) carries oxygen to every cell of the tissues and organs and removes waste products from them. A normal healthy RBC is usually in the form of disc—like a doughnut—so that it is flexible enough to slip through small and large blood vessels to deliver oxygen to each organ. Sickle cell disease (SCD) is an inherited blood disorder, in which RBC looks like a sickle; these abnormal RBCs are inflexible and sticky and tend to stack one over another; as a result, they could not flow in the microvessels. The net result is lack of oxygen to the tissue causing severe pain (called crisis), or even organ damage. Over a lifetime, SCD can harm the patients' spleen, brain, joints, and so on. Since these abnormal cells experience greater friction with the walls and among themselves, they tend to burst apart (or hemolyze) with an average life span of 15–30 days, in comparison to 120 days of RBC of normal adult. In developed countries, with advanced medical care, the life expectancy for the SCD patient is about 50 years, otherwise 20 years in less developed countries.

A single red blood cell contains approximately 270 million molecules of hemoglobin proteins. SCD is due to inherited two abnormal hemoglobin genes, one from each parent. If only one gene is abnormal, the subject is said to have sickle cell trait (SCT), who will not have marked or serious medical symptoms; but when a subject with SCT marries another subject of SCT, each of their child has twenty five percent probability of having SCD and twenty five percent being normal and fifty percent probability of SCT. When this happens, these children suffer throughout their lives, and they would transmit SCT or SCD to the progeny. Because of these, many countries like Mediterranean region of Europe, Middle East, and South East Asia have made premarital screening mandatory for sickle and thalassemia diseases. This has reduced the incidence of these diseases significantly, with the classic example being Turkey [1, 2].

As of 2013, about 3.2 million people have SCD and 43 million have SCT (about 14 times more) globally. The western and southern region of the Kingdom of Saudi Arabia has very high incidence of SCD (2%) and SCT (15%) [3]. The most common conventional method of screening for any inherited blood disorder is clinical examination followed by CBC and electrophoresis [4–6], or occasionally HPLC [7]. Out of these, electrophoresis is based on the difference in the movement of different hemoglobins, under the influence of a given electric field, and HPLC is based on the movement of different hemoglobins in a given gravitational field, as they pass through certain adhesive materials.

Above two most common instruments are bulky, slow, and expensive. The new technique being proposed by this report is based on the spectral analysis of biomolecular fluorescence indicative of SCT and SCD due to disproportionate concentration of amino acids and coenzymes. When light of particular wavelength interacts with a biomolecule, it sends back responses in terms of signals of Raman scattering and fluorescence which are fingerprints of a particular molecule. By measuring them, different types of biomolecules could be identified and quantified. The concentrations of such biomolecules go out of proportion when a certain biological imbalance sets in. This is the essence of disease detection by molecular fluorescence. This work is an extension of similar spectral analysis of blood from patients of cancer or many other blood disorders. [8–13]. The biggest advantage of fluorescence is it is very sensitive, and the instrumentation could be made compact and transportable (within 5 kg).

## 2. Material and Methods

### 2.1. Study Population

A total of 90 samples were used for this study; the distribution of which is given in Table 1. All confirmed cases of SCD and SCT samples were received from King Khalid University Hospital (KKUH), Riyadh, Saudi Arabia (KSA). All healthy control samples were collected from staff; colleagues of KKUH and King Saud University, KSA; and Hemoglobinopathy Diagnosis Center of Mediterranean Blood Diseases Foundation, Antalya, Turkey. The patients and healthy persons were informed about the investigation, and proper consents were obtained. The Institutional Review Board approval (E–17-2267 dated 27 March 17) had been obtained for this project. The normal control group selected had no history of sickle, thalassemia diseases, thalassemia trait, iron deficiency, diabetes mellitus, alcoholism, or any other blood-related illness.

### 2.2. Spectrofluorometer Analysis

The investigation was carried on two types of samples, namely, blood plasma and RBC of SCD, SCT, and subtypes of SCD always in comparison with the normal control.

### 2.3. Blood Samples

For normal controls, 5 ml of arterial blood sample (age, sex adjusted) was collected from each control using arterial catheter into an EDTA tube. This tube was gently rocked 5 times for thorough mixing of EDTA and whole blood. The sample was centrifuged at 1760*g* for 15 minutes; after that, the clear, pale greenish yellow plasma of 1.5 ml was pipetted out in the quartz cuvette for spectrofluorometric analysis. Such blood plasma sample was subjected to synchronous fluorescence excitation spectral (SXS) analyses, as such without any other treatment.

Next, the buffy coat which contained most of the white blood cell (WBC) and platelets was removed and discarded. Then, one ml of the thick erythrocytes was pipetted out into a serial tube, and then 2 ml of analytical grade acetone was added. Proper care was taken to ensure that the formed elements did not develop lumps. After thorough mixing to enable the acetone to extract fluorophores within and around the cells, the sample was centrifuged again (1760*g* for 15 minutes). The resulting supernatant was subjected to fluorescence emission spectral (FES) analysis at an excitation wavelength of 400 nm. The same protocol was used to do blood sample collection, preparation, and spectral analysis for samples obtained from subjects of SCT and its subsets.

### 2.4. Instrumentation

The instrument used was a spectrofluorometer (PerkinElmer Luminescence LS 55) capable of collecting excitation, emission, and synchronous spectra in the 200–800 nm range. An excitation and emission slit width of 10 nm and scan speed of 1000 nm/min were used. Each sample was placed in quartz cuvettes and illuminated by a specified wavelength of light with a 10 nm spectral width and a spot size of 3 × 2 mm. There are a few variants in fluorescence spectroscopic technique [14, 15]. Out of these, only fluorescence emission spectra (FES) and synchronous excitation spectra (SXS) are employed for this report, and further details of these are available in earlier publication [8–13].

## 3. Results

**Figure 1:**
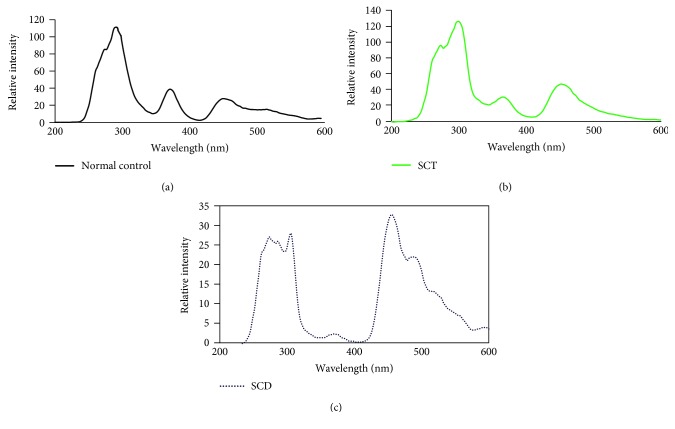
(a) The synchronous fluorescence excitation spectra (SXS) of normal plasma. The four peaks are at 275 nm due to tyrosine, (here only a shoulder); at 290 nm due to tryptophan; at360 nm due to NADH, and at 460 nm due to FAD. (b) The synchronous fluorescence excitation spectra (SXS) of SCT plasma. (c) The synchronous fluorescence excitation spectra of SCD plasma.

Figure 1(a) represents the typical synchronous excitation spectra (SXS) of plasma of a control male of age 25. This shows three major fluorescence excitation peaks: one at 290 nm (due to the amino acid tryptophan), the next at 360 nm (due to the enzyme, nicotinamide adenine dinucleotide (NADH)), and the third at 460 nm (due to another, enzyme flavin adenine dinucleotide (FAD)). A set of ratio parameters is defined to compare the spectra of control with the disease: *R*_1_ = *I*_290_/*I*_360_: here, *I*_290_ is the intensity at 290 nm and *I*_360_ is the intensity at 360 nm; another ratio is *R*_2_ = *I*_460_/*I*_360_: here, *I*_460_ is due to intensity at 460 nm; there is one more ratio *R*_3_ = *I*_275_/*I*_290_: here, the band at 275 appears only as a shoulder for control but becomes prominent for a certain type of patients. For the normal control, the above ratios are *R*_1_ = 2.5 ± 0.3, *R*_2_ = 0.6 ± 0.2, and *R*_3_ = 0.7 ± 0.1.

Figure 1(b) shows the SXS for the plasma of a subject of sickle cell trait (SCT) (HbS range 25%). Here again, the spectra has three peaks at 290, 360, and 460 nm, but the ratios *R*_1_, *R*_2_, and *R*_3_ are different. For this particular case of HbS = 25%, *R*_1_ = 4.5, *R*_2_ = 1.3, and *R*_3_ = 0.8. Note also that the peak intensity is only 120 (compared to 110 of the control as shown in Figure 1(a)). That is, all the ratio parameters are enhanced but the peak intensities are almost same. On the other hand, for a subject of sickle cell disease (SCD), with HbS range = 80%, *R*_1_ = 10, *R*_2_ = 12, and *R*_3_ = 1. There is dramatic increase in all ratio parameters; at the same time, the peak intensity is only 25. As shown in Figure 1(c), the SCD patient has fourfold decrease in the essential amino acids particularly tryptophan at 290 nm and tyrosine at 275 nm, which means this patient will have poor stamina and also the RBC undergoes premature hemolysis (high values of *R*_2_).

**Figure 2:**
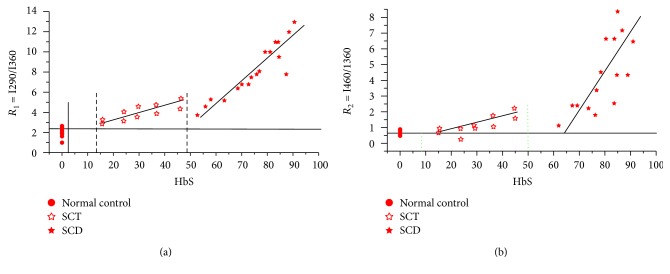
(a) The ratio of *R*_1_ for SCT and SCD. (b) The ratio of *R*_2_ for SCT and SCD.

Figure 2(a) shows variation of *R*_1_ as a function of the concentration of HbS. It can be seen that as HbS concentration increases, there is a gradual increase in *R*_1_ from the normal level of 2.5 to 5; but after a level of HbS = 50%, the ratio *R*_1_ has a steep dramatic increase and goes up to 14. The two regions SCT (HbS < 45%) and SCD (HbS > 45%) could be fitted only by two independent straight lines with two different slopes. The slope *m*_1_ = 0.08 for ST and *m*_2_ = 0.27 for SCD and *m*_2_/*m*_1_ = 3.4.

Figure 2(b) shows the variation of the ratio parameters *R*_2_ as a function of HbS. Here again, the ratio *R*_2_ increases linearly with HbS, but with two slopes for two regions, namely, SCT and SCD. The slope *m*_3_ = 0.07 for SCT and *m*_4_ = 0.28 for SCD. It is important to note that *m*_4_/*m*_3_ = 4.

There are three major classifications of inherited blood disorders: (1) sickle cell anemia (trait and disease), (2) thalassemia (trait and disease), and (3) G6PD (trait and disease) and a host of combinations of them. However, we had confined our investigation only to four categories, based on prevalence rates of these ramifications of blood disorder: SCT, SCD, SCD + *β*TT, and SCD + *β*TD.

**Figure 3:**
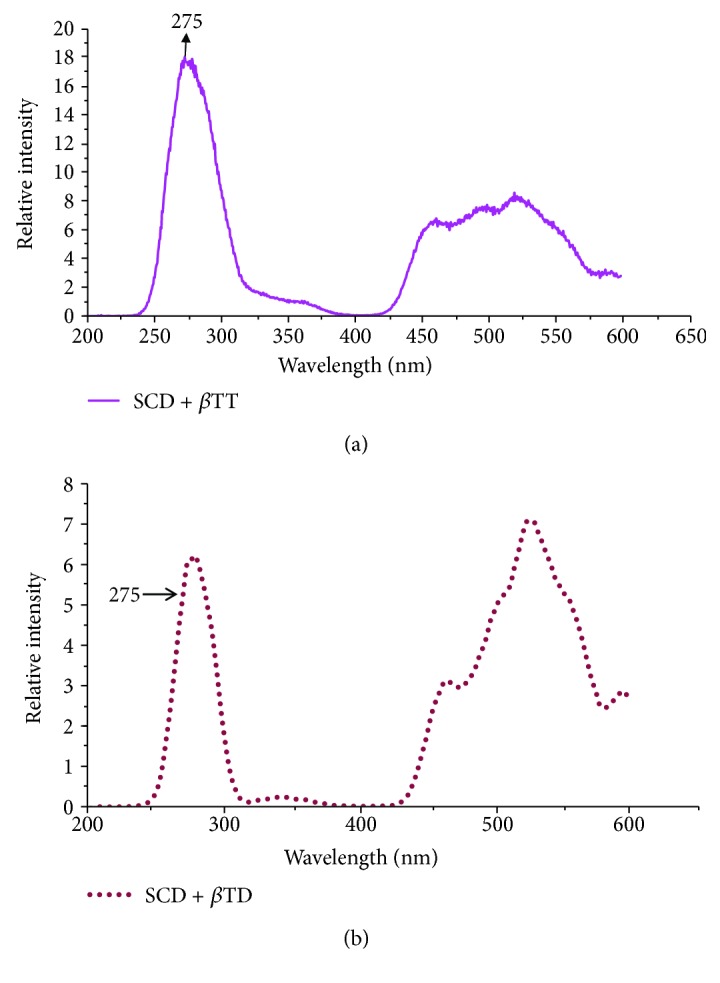
(a) The synchronous fluorescence excitation spectra of plasma of SCD + beta Thal trait. (b) The synchronous fluorescence excitation spectra of plasma of SCD + beta Thal disease.

Figure 3(a) gives the SXS of SCD + *β* Thal trait (*β*TT). The spectrum is similar to that of SCD, but with a few conspicuous spectral fingerprints of *β*TT. The first one is the first peak appearing at 275 nm, not at 290 nm as in the case of SCT or SCD. Secondly, the intensity of the left peak is only 80% of SCD. Thirdly, the intensity at 360 nm is very low and that at 460 nm is very high, so that *R*_1_ = 14 and *R*_2_ = 5; note that *R*_3_ = 1.1.

The situation becomes even worse, for the patient with SCD + *β*TD as shown in Figure 3(b). The left peak is again at 275 nm and the intensity is far less. Note also that the ratios *R*_1_ and *R*_2_ go awry with *R*_1_ = 18 and *R*_2_ = 14; *R*_3_ = 1.3 indicates the impact of *β* Thal disease on sickle disease.

**Figure 4:**
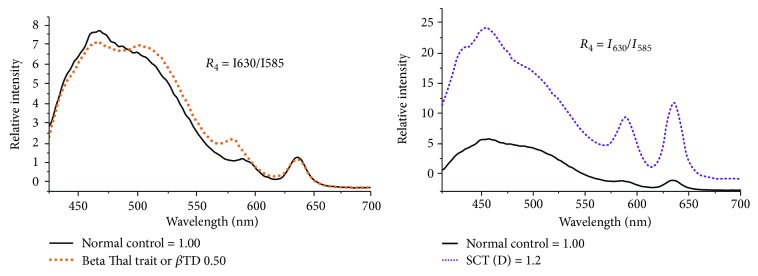
Black color spectrum is the normal control, red color spectrum is beta Thal, and purple color spectrum is sickle disease.

**Figure 5:**
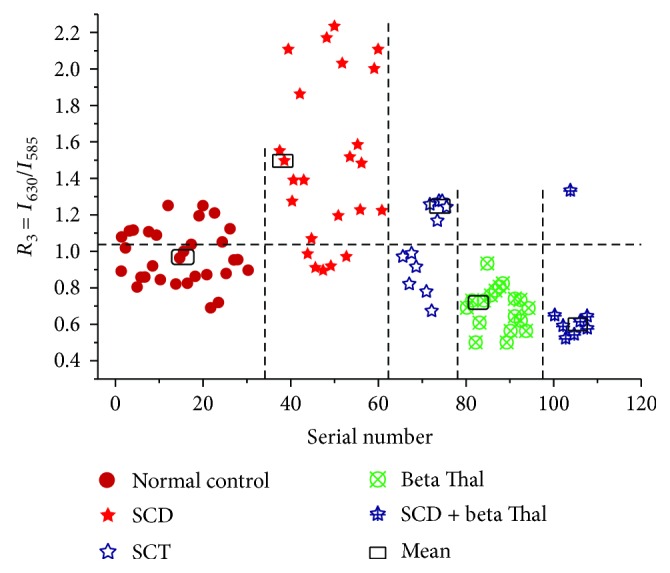
The scatter plot for the ratio *R*_4_, obtained from CC extracts, for a certain set of patient's blood disorder.

In addition to dramatic variation in the spectral features of plasma, the acetone extract of cellular components indicates conspicuous differences.  Figure 4(Black color spectrum) shows the typical fluorescence emission spectra (FES) of acetone extracts of cellular components of the normal control. There are three main fluorescence bands: at 480 nm (due to NADH), at 585 nm (due to basic porphyrin), and another at 630 nm (due to neutral porphyrin). A ratio is defined as *R*_3_ = *I*_630_/*I*_585_ = 1 for normal control but varies from 1.1 to 1.6 for SCT and SCD, but its range is 0.0.3 to 0.7 for *β*TD.  Figure 5 indicates the scatter plot for the ratio *R*_4_ for samples of the normal controls and variants of sickle diseases.

## 4. Discussion

Inherited blood disorders like sickle cell anemia and thalassemia are widespread chronic diseases affecting a large segment of people particularly in the Mediterranean and sub-Saharan belt. Though the reasons for such disorders are unknown, consanguineous marriage and malarial infection are conducive factors for the incidence and propagation from one generation to another.

In sickle cell hemoglobin, there is a genetic substitution of valine for glutamic acid at the sixth position in the two *β* chains which easily supports an intracellular H-bonding. This changes the conformation to allow molecular stacking and polymerization more so in the deoxygenated form. Such sickle erythrocyte leads to vasoocclusion and hemolysis. When the percentage of the abnormal hemoglobin HbS number is less than a certain clinically defined value (HbS < 45%), such damages are limited and often the carrier subjects will not have marked health issues. On the other hand, when HbS > 45%, the subject becomes a chronic patient and ends up with enduring disease [16, 17]. If this kind of hemoglobin is less than 45%, the subjects are termed as a trait or carrier and do not need repeated medical treatment. This is clearly shown up in a spectroscopic technique which just measures a set of fluorescent biomolecules. Out of a host of fluorescent biomolecules, tyrosine and tryptophan are essential amino acids essential for tissue growth and wellness. As shown by Figures 1(a) and 1(b), the level of the above two amino acids is almost like that of the normal control indicating the fact that SCT with HbS = 25% is almost as healthy as the normal control. This is not true for SCD since the amino acids (as evidenced by their excitation peaks at 275 and 290 nm) are few times less indicative of greater fatigue and poorer health conditions.

The other two fluorescent biomolecules measured in SXS of plasma are NADH and FAD. These two are coenzymes involved in any redox process and essential functions of any cell including RBC. The normal RBC has an average life span of 120 days, after which it decays and gets excreted. In the decay process of RBC, the above two enzymes are involved, and for the decay of each RBC, two NADH molecules are used up to produce one FAD. Hence, the ratio *R*_2_ = *I*_460_/*I*_360_ is a measure of decay of RBC. This ratio is about 0.6 for normal but goes up to 2 for SCT. From the above figures, it could be seen that the RBC of SCT may have less life span (of 80–100 days). This ratio is about 10 or more for SCD indicative of much shorter life span of 20–40 days. It is because of the abnormal decrease in NADH content in the plasma that the ratio R1 also is unusually high for SCD.

The blood disorder *β* Thal anemia is indicated by HbA2 going beyond the normal 3.5. In an earlier report, detailed investigations show that RBC undergoes fast decay and each RBC has lower oxygen content for these patients [18–20]. *β* Thal is due to a defective *β* segment of the hemoglobin which often leads to enhanced HbS too. Hence, combined sickle + beta Thal problems are much more prevalent than pure sickle cell anemia or disease. In fact, in a random collection of samples of sickles, we found that almost 70% have *β* Thal + SCA (or D). When this happens, the situation gets worse and the hemolytic rate seems to have a compounded faster rate than them in isolation. This is evident from Figures 3(a) and 3(b). In these cases, essential amino acids like tyrosine and tryptophan are deplorably less and hemolytic by-products are alarmingly high.

Apart from the measurement of biomolecules of plasma, the cellular component measurement indicated that for the normal control, the neutral porphyrin with a peak at 630 nm and that 585 nm are in equal proportion (with a kind of check and balance). But in the case of *β* Thal trait or disease, 630 nm is down by 40% (*R*_4_ = 0.6). This only means that the oxygen content of the RBC is low for *β* Thal; surprisingly, this ratio is higher than the control by 20 to 50% for the SCT and SCD. In spite of it, the *R*_4_ values could not come up beyond 0.7 for subjects with compounded sickle (SCD + beta Thal) which means beta Thal has a stronger impact on subjects than SCD in oxygen content.

The above two sets of results, one for plasma and the other for acetone extract of RBC, lead us to the following hypotheses.

The subjects of *β* Thal trait would suffer mostly due to less oxygen content in each RBC but not due to hemolysis, but *β* Thal disease subjects suffer due to less oxygen content and also due to hemolysis because of the inherent weakness of the RBC. In contrast, the SCT subject with HbS < 45% has enough oxygen content in each RBC but suffers due to mild hemolysis as the abnormal Hb undergoes friction along the microvessels. The SCD subject undergoes greater mechanical damage due to the greater friction and fragility, though the oxygen content of each RBC is comparable or even better than normal RBC. This would mean that for SCT and SCD subjects, less number of RBC reaches the distant organs due to misshape of each RBC. Perhaps the internal feedback mechanism for SCD or SCT subjects could make each RBC 20 to 40% more enriched with oxygen content. All these are hypothesis borne out by this spectral investigation and must be proved by independent techniques.

## 5. Conclusion

In a novel technique which measures the relative concentration of a set of fluorescent biomolecules, it has been possible to give some insights into the mechanism of four of the most common blood disorders in the Gulf region. The study shows that sickle cell patients suffer mostly due to mechanical damage of abnormal RBC though each RBC is sufficiently rich in oxygen content, but Thal patients have inherent weakness in each RBC. Also, SCT, SCD, SCD + *β*TT, and SCD + *β*TD have shorter and shorter life span through we could not quantify them at this point of investigation.

## Figures and Tables

**Table 1:** Distribution of subjects.

Normal	SCT	SCD	*β*TT + SCD	*β*TD + SCD
*N* = 30	*N* = 10	*N* = 25	*N* = 10	*N* = 15
18 male, 12 female	6 male, 4 female	14 male, 11 female	5 male, 5 female	7 male, 8 female
Median age 26 for male and 19 for female	Median age 26 for male and 19 for female	Median age 20 for male and 16 for female	Median age 20 for male and 14 for female	Median age 18 for male and 13 for female

## Data Availability

The data used to support the findings of this study are available from the corresponding author upon request.
